# Mesenchymal properties of SJL mice-stem cells and their efficacy as autologous therapy in a relapsing–remitting multiple sclerosis model

**DOI:** 10.1186/scrt524

**Published:** 2014-12-12

**Authors:** Carmen Marin-Bañasco, Margarita Suardíaz García, Issac Hurtado Guerrero, Rafael Maldonado Sánchez, Guillermo Estivill-Torrús, Laura Leyva Fernández, Oscar Fernández Fernández

**Affiliations:** Unidad de Gestión Clínica Inter-centros de Neurociencias, Laboratorio de Investigación y Servicio de Neurología. Instituto de Biomedicina de Málaga (IBIMA), Hospitales Universitarios Regional de Málaga y Virgen de la Victoria, 29009 Málaga, Spain

## Abstract

**Introduction:**

Mesenchymal stem cells (MSCs) are a multipotent population of adult stem cells, which may represent a promising therapeutic approach for neurological autoimmune diseases such as multiple sclerosis. The mouse is the most used species for obtaining and studying the characteristics of MSC and their potential as autologous transplants in pre-clinical models. However, conflicting data have been published disclosing intraspecies variations. The choice of the mouse strain and the tissue source appear, among others, as important factors in the experimental application of MSCs.

**Methods:**

Adipose tissue-derived MSCs obtained from the SJL/JCrl mouse strain (SJL-AdMSC) have been cultured for a long time (from passage 0 up to 15) under controlled experimental conditions, and their growth rate, morphology, stromal and haematopoietic marker expression profiles and differentiation capacity towards adipocytes, osteocytes and chondrocytes have been determined. Moreover, their preclinical efficacy has been assessed by autologous transplant in relapsing-remitting experimental autoimmune encephalomielitis (RR-EAE)-induced SJL mice (a well established mice model for the study of RR-multiple sclerosis).

**Results:**

We demonstrate that SJL-AdMSCs show the same fibroblastic shape, growth rate, profile of markers expression and multipotency described for MSCs in every passage evaluated (up to passage 15). Additionally, SJL-AdMSCs ameliorate the RR-EAE course, suggesting that they could modulate disease progression. Moreover, their features studied are fully comparable with the standardized Ad-MSCs obtained from the C57BL/6 mouse strain, which strengthens their use in cell therapy.

**Conclusion:**

SJL-AdMSCs might be a suitable source of Ad-MSCs for studies related to the properties of MSCs and their application as promising therapeutic tools in autologous transplants in experimental medicine.

## Introduction

Mesenchymal stem cells (MSCs) are a multipotent and heterogeneous subset of adult stromal stem cells present in many tissues. Traditionally, the study of the therapeutic potential of these cells has been oriented to their application in tissue repair and regeneration, due to their differentiation capacity and transdifferentiation into different cell lines [1, 2]. However, over the past decade, MSCs have been shown to possess a broad spectrum of immunoregulatory capabilities affecting both adaptive and innate immunity. This has been proven by *in vitro* experiments and their effectiveness when transplanted into animal models of autoimmune diseases [3–5]. MSCs therefore represent a promising tool as cell therapy, not only for regenerative medicine but also for modulation of the immune system [6–9].

The biological characteristics with which cell populations must comply to be defined as MSCs are clear, regardless of the tissue of origin [10, 11]. In recent years, several studies have described isolation protocols, culture, expansion, phenotypic and functional characterization of human and animal MSCs derived from different tissues [12–17]. However, although these cells are assumed to be similar, contradictory results have been obtained revealing interspecies/intraspecies variations regarding cell morphology, cell survival and senescence [18], cell proliferation, surface marker profile, growth kinetics [9, 12, 15], differentiation capacity [3, 16], gene expression [13], genetic instability or even malignant transformation [14], among others. All these conflicting data may be a result of a multitude of factors that have been shown to play an important role in these biological characteristics, such as cell isolation protocols, culture medium formulation [9, 15], cell density of the starting culture [17], different culture time and expansion conditions [9, 14, 15], species and/or tissue source from which MSCs are obtained [12, 13, 15, 19–21] and experimental approaches [15, 16, 19, 22], which vary among researchers. It is currently hard to compare the results from different laboratories, which highlights the necessity to define the best isolation site and expansion methods for MSCs.

The ultimate goal in MSC research is to treat human patients at the clinic with their own MSCs, since autologous transplantation provides two main theoretical advantages: a minimization of the risk of transmission of infectious diseases and an increased efficiency in the absence of rejection by the patient’s immune system [23]. The only way to truly imitate this strategy in animal studies is by transplanting animals with MSCs derived from a peer animal of the same species and strain [24]. The mouse is a very suitable experimental system to study MSC biology and their potential clinical benefits. Although several reports have been published comparing MSCs from different mice strains and tissues, there is currently no consensus in the culture conditions nor in the description of the disagreements existing between these cells [12–15, 19–21]. These features limit greatly the ability to test the cells in the large number of interesting murine models now available for autoimmune and neurodegenerative diseases and/or for transgenic mice, in which the mouse genetic background largely influences the experimental model [25–27]. In this sense, the choice of the mouse strain and the tissue source appear as important factors in the experimental application of MSCs.

Here we have made a comparative characterization, under the same experimental conditions, of adipose tissue-derived mesenchymal stem cells (Ad-MSCs) from two different mouse inbred strains, C57BL/6 and SJL/JCrl. C57BL/6 is one of the most used mouse strains in basic and preclinical research, and represents a general multipurpose model, which is also frequently used in diet-induced obesity studies, transgenic/knockout model development and safety and efficacy drug testing, among others. The SJL/JCrl mouse strain is less common but not the least, being especially important in studies focused on retinal degeneration [28] and also for developing transgenic models [29].

Furthermore, both C57BL/6 and SJL/JCrl strains provide a useful experimental tool, since they both carry the susceptible genetic backgrounds more commonly used to develop experimental autoimmune encephalomyelitis (EAE) in mice. EAE is an animal model of T-cell-mediated central nervous system demyelination and represents the most frequently used animal model for multiple sclerosis (MS) research. MS has several clinical forms, including initial attacks of optic neuritis, episodes of relapsing and remitting paralysis and sensory deficits, and more progressive deteriorations [30]. Although there is not a single animal model that can capture the entire spectrum of heterogeneity of human MS and its variety in clinical and radiological presentation, over the last decades useful and relevant EAE animal models that share clinical and neuropathological changes with human disease have been developed [30–34]. Thereby, chronic progressive experimental autoimmune encephalomyelitis (CP-EAE) models, in which neurological deficit advances in a progressive course or becomes chronic over time without clear episodes of relapse and remission, approach the model of the primary progressive form of MS in humans. Otherwise, by mimicking the relapsing–remitting MS progression form, the relapsing–remitting experimental autoimmune encephalomyelitis (RR-EAE) model facilitates the study of the underlying mechanisms of relapses and remittances [30, 35]. This wide variety of clinical EAE courses is determined by multiple factors used to induce the model, such as the animal’s sex, the strain genetic background, the antigen used for immunization, and so forth. Whereas the C57BL/6 mouse strain is the most widely used to develop myelin oligodendrocyte glycoprotein 35–55-induced CP-EAE, the SJL/JCrl strain is usually preferred to set the myelin proteolipid protein 139–151-induced RR-EAE model [25, 35–39].

Current MS cell-based therapeutics, as in many other diseases, require validating in animal models, replicating the possible pathological courses as well as the characterization of many factors in order to exclude possible variability sources in the results. The Ad-MSC cultures obtained from the C57BL/6 mice strain (C57-AdMSCs) had been characterized previously, and based on their immunophenotype and growth potential they are considered a suitable source to establish a mouse model for obtaining Ad-MSCs in preclinical studies [12, 13, 15, 16]. Moreover, many preclinical works have reported their immunomodulatory effects in EAE models, which makes them a valuable tool for stem cell-based therapy in immune-mediated diseases [4, 5, 40]. In contrast, to the best of our knowledge, analogous studies have not been performed with Ad-MSCs from the SJL/JCrl mouse strain (SJL-AdMSCs). The characterization of SJL-AdMSCs is therefore essential for preclinical studies of autologous transplantations in this and other diseases.

In this study we demonstrate that, under our experimental conditions, SJL-AdMSCs can be isolated, expanded and maintained in culture for a long period of time with a stable growth rate in a highly reproducible manner. These cells also fulfill the characteristics that define MSCs regarding morphology, immunophenotype and differentiation capacity. In addition, we developed a preclinical study showing that SJL-AdMSCs reduce disease severity in RR-EAE-SJL/J-induced mice. Our findings therefore suggest that the SJL/JCrl mouse strain may be an adequate source for obtaining Ad-MSCs for use in preclinical research of autologous cell therapy in autoimmune diseases.

## Methods

### Mice

All experiments were conducted using adult (6 to 8 weeks old) female mice from SJL/JCrl (H2^s^) and C57BL/6 (H2^b^) mouse inbred strains, purchased from Charles River Laboratories International, Inc. (Barcelona, Spain). These animals were housed in clear plastic cages under specific pathogen-free conditions in accordance with institutional guidelines. A controlled temperature (23 ± 1°C), a 12-hour: 12-hour light/dark cycle and free access to food and water were set. The study was approved by the Ethics and Research Committee of the Carlos Haya Regional Hospital, and all experiments were performed in compliance with the European Animal Research Laws (European Communities Council Directives 2010/63/EU, 90/219/EEC, Regulation (EC) No. 1946/2003) and the Spanish National and Regional Guidelines for Animal Experimentation and Use of Genetically Modified Organisms (Real Decreto 53/2013 and 178/2004, Ley 32/2007 and 9/2003, Decreto 320/2010).

### Isolation, culture and expansion of adipose-derived mesenchymal stem cells

After sacrificing the mice by cervical dislocation, adipose tissue fragments were obtained from subcutaneous abdominal fat tissue. The isolation of murine Ad-MSCs from both strains was performed attending to slight modifications from a previously described protocol [4]. Briefly, the adipose tissue fragments were washed in Dulbecco's modified Eagle's medium, mechanically dissociated and subsequently digested in serum-free Dulbecco’s modified Eagle’s medium (Sigma-Aldrich Química S.L., Madrid, Spain) supplemented with collagenase A (1 mg/ml; Worthington, NJ, USA), gently shaking for 2 hours at 37°C. After tissue disaggregation, the cellular suspension was filtered through a 100 μm cell strainer (Becton Dickinson, Franklin Lakes, NJ, USA) to remove debris and centrifuged twice to wash the cells. The resulting pellet was resuspended in a specific growth medium for murine MSCs, Complete MesenCult® Medium – that is, MesenCult® Basal Medium supplemented with MESENCULT® Mesenchymal Stem Cell Stimulatory Supplements (both STEMCELLS Technologies, Grenoble, France) – streptomycin and penicillin (PAA Laboratories GmbH, Pasching, Austria), and thereafter seeded in culture plates (1 × 10^6^ cells per 9.6 cm^2^ dish) until the cell culture reached 100% confluence.

The Ad-MSCs were then trypsinized and expanded *in vitro* with the aforementioned supplemented medium, replacing it every 2 days during the expansion phase. The cells were plated at 6 × 10^3^ cells/cm^2^ in cell culture dishes (450,000 cells per 75 cm^2^ dish) and cultured in a 37°C incubator with a 5% carbon dioxide atmosphere. Every time they reached 80% confluence, the cells were trypsinized, harvested and counted. The cells were then subcultured at the same cell density from one up to 15 passages.

We performed four replicates for each inbred strain. Each replicate was a mixture of Ad-MSCs obtained from two mice.

### Biological characteristics of the adipose tissue-derived MSCs

Cell morphology was determined by direct visualization with a trinocular inverted phase contrast microscope (Leica DMIL LED; Leica Microsystems, Inc., Barcelona, Spain). Images were captured at 10× magnification.

The population growth rate was calculated by determining the doubling time (period of time required to double the size of the population) using the Schwartz formula [41, 42]:


where *DT* is the cell population doubling time, *t* is the time lapse between two measurements (two consecutive culture passages), *D*_*t*_ is the number of cells at the final measurement (in passage *X* + 1), and *D*_*0*_ is the number of cells at the initial measurement (in passage *X*). During the expansion phase, to calculate the DT, the number of grown cells was obtained using the conventional Trypan Blue vital stain methodology in all passages. Data are expressed in hours, and are presented as the mean ± standard error of the mean (SEM) of four DT values obtained from Ad-MSC cultures of each strain.

### Adipose tissue-derived MSC surface marker expression: fluorescence-activated cell sorting analysis

For phenotyping, the cells harvested from each passage (passages 3 to 15) were aliquoted (1 × 10^5^ cells/vial), washed with saline and stained using fluorescein isothiocyanate, phycoerythrin, phycoerythrin–cyanine Dye 7, allophycocyanin orallophycocyanin–Cy7 mouse monoclonal antibodies against mouse stromal markers CD44, CD106 (Becton Dickinson) or CD90.2 (eBioscience, San Diego, CA, USA), as appropriate, at 4°C for 30 minutes. In addition, the absence of hematopoietic markers was assessed by staining cells with monoclonal antibodies against mouse CD45 (Miltenyi Biotec S.L., Madrid, Spain), CD14 and CD34 (Becton Dickinson). Isotype-matched antibodies were used as controls. We also observed the cells’ size and granularity by analyzing the forward-scatter signal and side-scatter signals during the culture time. The forward scatter signal is expressed in arbitrary units.

After being washed with phosphate-buffered saline, the cells were fixed in phosphate-buffered saline with 1% paraformaldehyde (PFA; Sigma-Aldrich Química S.L., Madrid, Spain). At least 5,000 events were collected for further analysis using a FACSCanto II cytometer and FACSDiva software (Becton Dickinson).

Summarized results are presented as arithmetic means ± SEM of four samples from each strain per culture passage. The Mann–Whitney *U* test was used to evaluate the differences between the mouse strains within each passage. *P* <0.05 was considered statistically significant.

### Adipose tissue-derived MSC differentiation assays

The adipogenic, osteogenic and chondrogenic potential was tested, in duplicate, in Ad-MSC populations from both strains at passages 7 and 15, according to the following protocols.

Briefly, Ad-MSCs were seeded at 6 × 10^4^ cells per well in a 24-well tissue culture plate (3 × 10^4^ cells/cm^2^) using Complete MesenCult® Medium. After they reached 80% semiconfluence, induction was carried out by replacing the growth medium for the supplemented MSC Adipogenic or Osteogenic Differentiation Medium (Ready-to-use) (PromoCell GMBH, Heidelberg, Germany) as appropriate, which contained all of the growth factors and supplements necessary for an optimal differentiation, streptomycin and penicillin. The media was carefully replaced every 2-3 days for 14 or 21 days, depending on whether it was adipogenesis or osteogenesis. The acquisition of the adipogenic phenotype was then confirmed by staining the monolayers – previously fixed in 4% PFA, and preincubated in 60% isopropanol – with a 0.25% Oil Red-O solution (PromoCell GMBH) and counterstaining with hematoxylin and eosin. The Ad-MSC colonies which underwent adipogenic differentiation exhibited cells that contained numerous, variable-sized lipid vesicles. To observe osteogenic mineralization, the cultures were prefixed in 4% PFA, washed once with phosphate-buffered saline, and stained for 5 minutes at room temperature with 2% Alizarin Red S stain (PromoCell GMBH), pH 4.2, which stained cell calcium deposits. The excess stain was removed by several washes with distilled water.

For chondrogenic differentiation, the Ad-MSCs were plated at 1 × 10^5^ cells per well in a 96-well U-bottom suspension culture plate using Complete MesenCult® Medium. Spheroids were spontaneously formed within 24 to 48 hours. After this period, the medium was removed and the cells were incubated for 21 days with supplemented MSC Chondrogenic Differentiation Medium (PromoCell GMBH) containing streptomycin and penicillin, carefully replaced every 2 to 3 days. The spheroids were then fixed in 4% PFA, frozen and sectioned 25 microns thick in a freezing microtome. The sections were stained with Alcian Blue 8GX (Sigma-Aldrich Química S.L.) used to stain acidic polysaccharides such as glycosaminoglycans in cartilages and other body structures, and counterstained with Nuclear Fast Red Solution (Sigma-Aldrich Química S.L.).

All differentiation images were captured at 10× or 20× magnification with a trinocular inverted phase contrast microscope (Leica DMIL LED) to acquire adipocytes and osteocytes images, and an Olympus BX41 Microscope with the Olympus DP70 camera associated (Olympus Iberia S.A.U., Barcelona, Spain) for chondrocyte images.

### Experimental autoimmune encephalomyelitis induction and clinical evaluation

CP-EAE was induced in C57Bl/6 (H2b) mice by subcutaneous immunization in the flanks with the myelin oligodendrocyte glycoprotein 35–55 (MEVGWYRSPFSRVVHLYRNGK) peptide (200 μg; GenScript, Piscataway NJ, USA) emulsified in complete Freund’s adjuvant containing 0.8 mg/ml heat-inactivated *Mycobacterium tuberculosis* (Becton Dickinson) at a final volume of 100 μl. The pertussis toxin (Sigma-Aldrich Química S.L.) was administered intraperitoneally by injection at the dose of 300 ng on days 0 and 2 post immunization.

To develop a RR-EAE, SJL/JCrl (H2s) mice were immunized using the same protocol described above but by performing the emulsion with the proteolipid protein 139–151 (HSLGKWLGHPDKF) peptide (300 μg; GenScript) as the antigen. Each animal also received a total of 300 ng pertussis toxin through two intraperitoneal injections on the immunization day and 48 hours later.

The clinical score was blindly registered according to a standard 0 to 5 scale [43]: 0, healthy; 0.5, flaccidity and partial paralysis of the tail; 1, limp tail; 1.5, weakness in one hind limb; 2, hind limb paresis; 2.5, partial hind limb paralysis; 3, total hind limb paralysis; 3.5, partial fore limb paralysis; 4, hind limb paralysis and body/front limb paresis/paralysis; and 5, moribund.

CP-EAE-induced animals were monitored over a 35-day period and RR-EAE-induced animals over 50 days. The endpoint evaluation included a variety of disease parameters, such as the disease incidence and mortality, the day of the disease onset, the maximal score, the mean score reached in chronic phase in CP-EAE animals, the duration and the mean scores of the relapsing periods in RR-EAE mice, and the cumulative score over experimental times.

### Treatment protocols

CP-EAE-induced and RR-EAE-induced animals from both mouse strains were randomly distributed in groups of 10 or 11 individuals to receive a unique administration of Ad-MSCs obtained from animals of their same strain (autologous transplant). All of the experiments were performed using Ad-MSCs at seven to 10 passages. Cells were injected intravenously through the vein of the tail when animals reached a clinical score between 0.5 and 1 (at 13 days post immunization (dpi) and 12 dpi for CP-EAE and RR-EAE models, respectively). For each injection, 1 × 10^6^ Ad-MSCs were resuspended in 400 μl saline as vehicle. The EAE-control mice group (*n* = 10 or 11) from both models received only vehicle (400 μl saline) by a similar protocol.

### Immunohistochemistry

EAE-induced animals were sacrificed by intraperitoneal administration of a lethal dose of pentobarbital. Fresh spinal cord extraction was performed at the chronification period (35 dpi) in CP-EAE-induced animals and at the peak of the second relapse (45 dpi) in RR-EAE mice.

The lumbar spinal zones were placed in 4% PFA (Sigma-Aldrich Química S.L.), cryoprotected in 30% (w/v) sucrose and cut in 40 μm cryostat sections. Histopathological analysis of spinal T-cell infiltrates and demyelination were determined by free-floating immunostaining using antibodies against the CD3 antigen (rabbit polyclonal; Abcam plc, Cambridge, UK) and the myelin basic protein antigen (rat monoclonal; Abcam plc), respectively. For their detection, Extravidin® peroxidase (Sigma-Aldrich Química, S.L.) and 3,3′-diaminobenzidine (Sigma-Aldrich Química, S.L.) methods were used as described previously [44].

Analysis were carried out over 4× and 10× images (three or four images per animal) using the microscope described above. Inflammatory infiltrates were determined by counting the number of labeled cells in 200 μm^2^. The area of demyelination, in which staining was negative for myelin basic protein, was expressed as the percentage of total myelinated area per section. Data are shown as the mean ± SEM of values obtained from six animals per experimental group.

### Statistical analysis

The data were expressed as the mean ± SEM and were analyzed with SigmaStat (SPSS Inc.,IBM Corporation, New York, USA). Student’s *t* test or the Mann–Whitney *U* test was used to compare pairs of groups from the different mice and cell cultures studied. Minimal statistical significance was set at *P* <0.05.

## Results

### Morphology and cell expansion characteristics of the adipose tissue-derived MSCs

**Figure 1:**
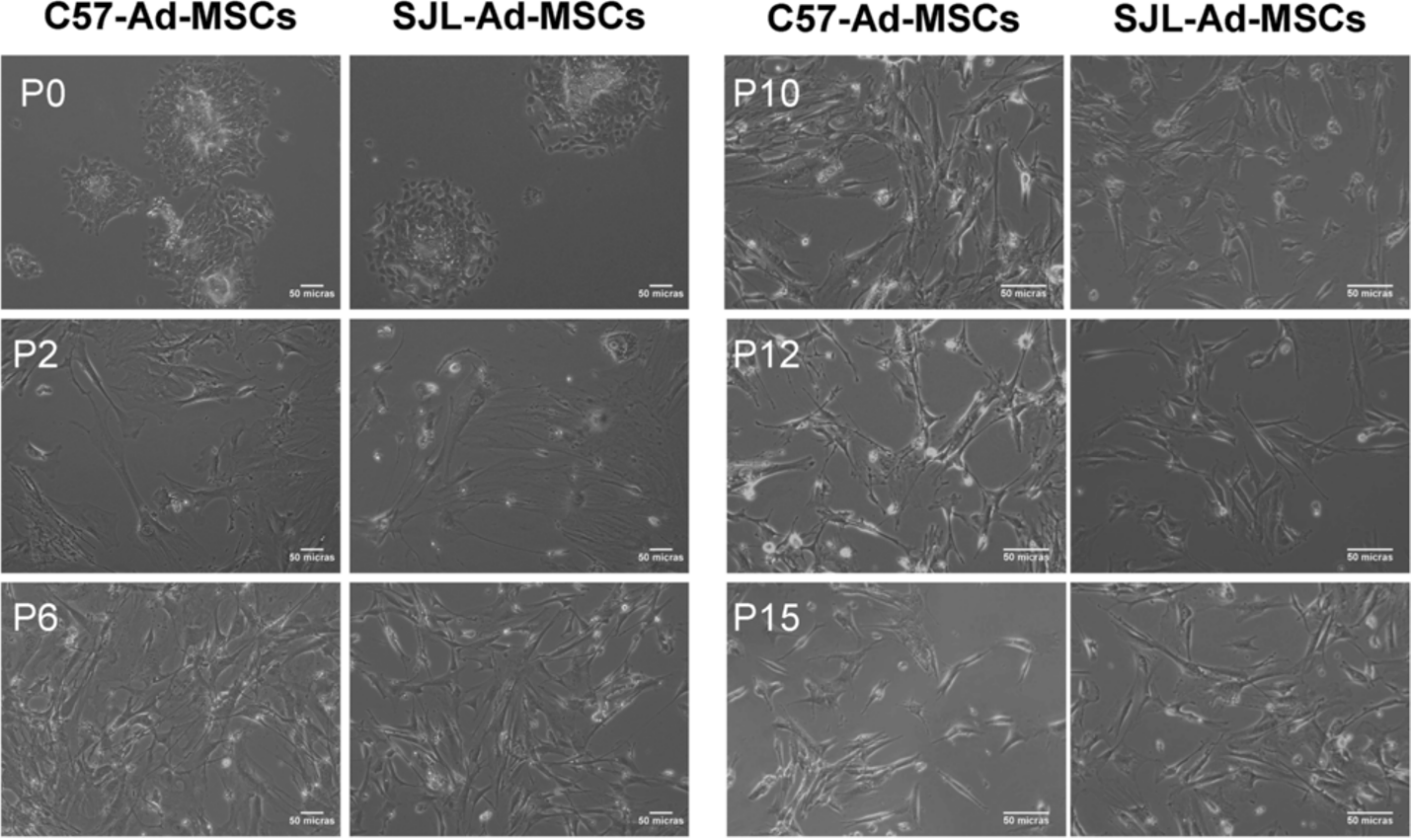
**Morphology of adipose tissue-derived mesenchymal stem cells isolated from SJL/JCrl and C57BL/6 mice strains.** Images of the C57-AdMSC and SJL-AdMSC cultures showing the morphology of the populations. Passage 0 (P0) images show cells with rounded morphology and colony growth. Images from passages 1 (P1) to 15 (P15) show that plastic-adherent C57-AdMSCs and SJL-AdMSCs have a fibroblastic morphology and expand primarily over the surface of culture dishes (original magnification 10×). Ad-MSC, adipose tissue-derived mesenchymal stem cell.

Ad-MSCs isolated from the SJL/JCrl mice strain (SJL-AdMSCs) and from the C57BL/6 mice strain (C57-AdMSCs) were seeded under the same conditions in culture plates and expanded during 15 passages, displaying a similar volume and cell morphology. The primary cultures (passage 0) of Ad-MSCs contained heterogeneous cell populations with different shapes. In this early phase, most of the cells were plastic adherent. They showed a rounded morphology and grew forming colonies, with no significant differences between the cells from both strains (Figure 1). However, in the following passages (from passages 1 to 15), where cells were seeded at a lower density than in primary cultures, the Ad-MSCs showed a fibroblastic-like shape and grew uniformly until confluence by colonizing the entire plastic surface, regardless of the mouse strain or culture passage analyzed (Figure 1).

To determine the potential growth rate of Ad-MSCs, we calculated the time (in hours) that the population took to double its number (DT) using the Schwartz formula [42]. In general, the average of the growth rate of Ad-MSCs increased gradually over the passages in both C57-AdMSC and SJL-AdMSC populations, as demonstrated by a decrease in the DT during cultivation (Table 1). Passages in which the DT stabilized at its minimum values were from 6 (15.5 ± 1.7) to 15 (16.5 ± 2.9) hours for the C57-AdMSC population, and from 7 (21.9 ± 2.8) to 15 (19.8 ± 3.4) hours for SJL-AdMSCs.

**Table 1:** **Doubling times of the adipose tissue-derived mesenchymal stem cell populations**

Passage	C57	SJL
1	44.7 ± 15.5	82.9 ± 16.24
2	60.2 ± 20.3	86.3 ± 43.70
3	69.6 ± 5.6	52.1 ± 20.4
4	64.8 ± 40.7	37.5 ± 1.2
5	39.0 ± 5.45	35.8 ± 5.8
6	15.5 ± 1.7	26.8 ± 4.6
7	22.5 ± 3.1	21.9 ± 2.81
8	25.4 ± 3.4	20.7 ± 1.39
9	27.9 ± 6.6	20.4 ± 1.95
10	26.9 ± 5.3	27.1 ± 1.96
11	15.4 ± 5.7	24.0 ± 2.9
12	16.1 ± 5.8	22.1 ± 3.2
13	14.7 ± 1.2	17.8 ± 1.9
14	18.6 ± 6.7	14.3 ± 0.5
15	16.5 ± 2.9	19.8 ± 3.4

Comparison of the growth potential between the C57-AdMSC (C57) and SJL-AdMSC (SJL) populations at passages 1 to 15. Population doubling times were calculated using the Schwartz formula (see text). Table shows the mean ± standard error of the mean of population doubling time values (in hours) obtained from the adipose tissue-derived mesenchymal stem cell (Ad-MSC) cultures of three replicate plates of each inbred mouse strain per passage.

### Adipose tissue-derived MSC phenotype characteristics

**Figure 2:**
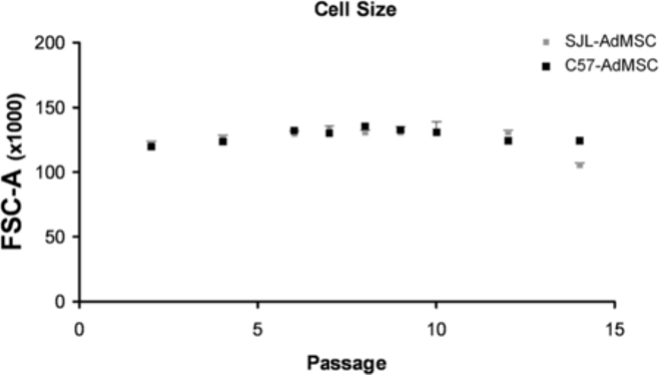
**Characterization of cell size through successive passages of the adipose tissue-derived mesenchymal stem cell populations.** Data represent the mean ± standard error of the mean of the forward-scatter signal (FSC, arbitrary units) values obtained from the adipose tissue-derived mesenchymal stem cell (Ad-MSC) cultures of four replicate plates of each inbred mouse strain per passage by flow cytometry.

Cells isolated from both mouse strains were analyzed in each culture passage by flow cytometry for their phenotypic profile, previously reported to be determinative for the MSCs [10]. Results showed that SJL-AdMSCs proliferated to clearly homogeneous populations exhibiting a forward scatter/side scatter signal plot of the median signal in the culture passages analyzed, which was attributed to the maintenance of the cell size (Figure 2) and granularity (data not shown) during *in vitro* cultivation. No differences were found after performing a *t*-test analysis comparing them with the C57-AdMSC population.

**Figure 3:**
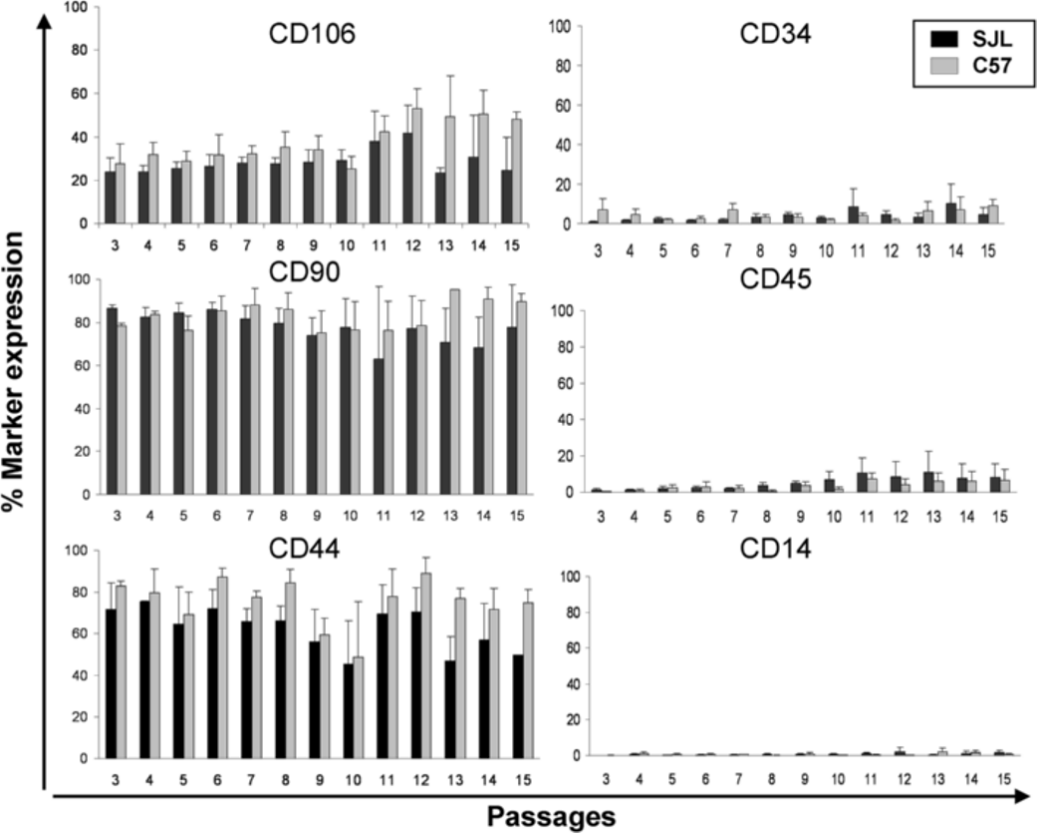
**Expression of stromal and hematopoietic markers in the SJL-AdMSC and C57-AdMSC populations by flow cytometry.** Graphs show the average (mean) of each CD marker expression percentage value ± standard error of the mean at passages 3 to 15. The Mann–Whitney *U* test was performed to compare the expression between cell cultures. No statistically significant differences were found between groups. Ad-MSC, adipose tissue-derived mesenchymal stem cell.

Less than 10% of the SJL-AdMSCs expressed the hematopoietic markers CD34, CD45 and CD14 in all of the passages tested (Figure 3). On the other hand, SJL-AdMSCs expressed variable levels of CD106 (VCAM-1), CD90.2 (Thy-1.2) and CD44 (receptor for hyaluronate and osteopontin) markers, with no statistically significant differences when compared with the C57-AdMSC population (Figure 3). In both strains, the moderate percentage of Ad-MSCs expressing the CD106 marker remained practically stable along the culture period with no significant differences, in agreement with the homogeneity exhibited in both cell populations. Regarding the CD44 and CD90 markers, the expression in the SJL-AdMSC population was high and remained stable in time through all of the passages. In C57-AdMSCs, this expression profile was similar, and kept until the end of the culture time.

### Adipose tissue-derived MSC differentiation potential

To validate the multipotentiality of the SJL-AdMSCs cultures, *in vitro* differentiation was induced into adipogenic, osteogenic and chondrogenic lineages in the middle and final phases of our experimental study (that is, passages 7 and 15), being the culture passages among those in which the cell growth rate stabilized at the maximum values.

**Figure 4:**
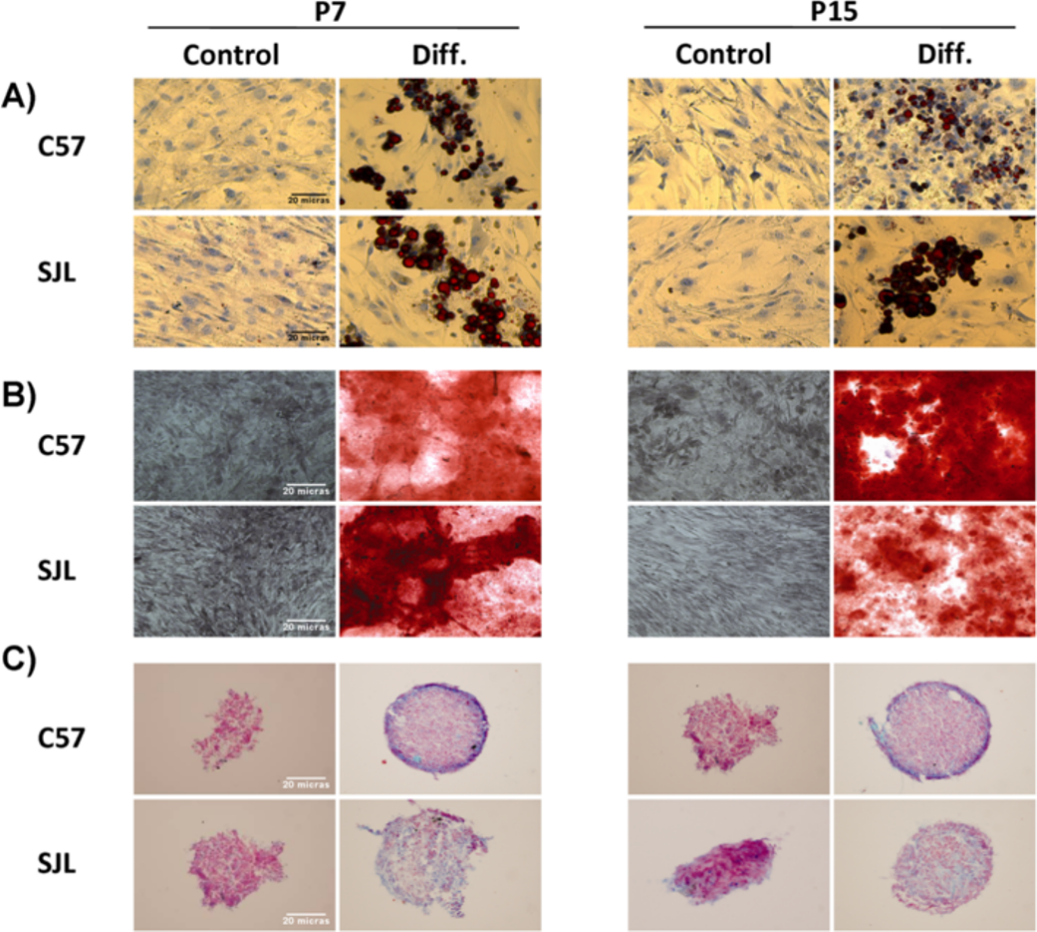
**Adipose tissue-derived mesenchymal stem cell differentiation. (A)** Adipocytes (original magnification, 20×), **(B)** osteocytes (original magnification, 10×) and **(C)** chondrocytes (original magnification, 10×). Images show the SJL-AdMSC and C57-AdMSC populations at culture passages 7 (P7) and 15 (P15). Cell cultures were maintained in the growth media (Control) and stimulated to differentiation (Diff.) by incubation with the specific media.

For adipogenic differentiation, Ad-MSCs were cultured in appropriate media for 16 days. The adipogenic potential of SJL-AdMSCs was similar in every passage evaluated, and showed no differences when compared with that of the C57-AdMSCs. After adipogenic induction, all of the Ad-MSC lines showed a high percentage of round cells with lipid vesicles occupying the cytoplasm, which is consistent with the phenotype of mature adipocytes (Figure 4A). No lipid droplets were observed in undifferentiated Ad-MSCs (control) in both passages analyzed.

SJL-AdMSC differentiation to osteocytes was evaluated by incubating cells with a specific osteogenic differentiation medium after 21 days. During this cultivation period, multiple layers of cells were formed. In a high percentage of the cases, these cells led to dense nodules from which radiated highly elongated spindle-shaped cells. Calcium deposition was demonstrated by Alizarin Red staining (Figure 4B). In both passages tested, SJL-AdMSCs and C57-AdMSCs showed a similar osteogenic potential, with no differences between passages.

The SJL-AdMSCs that underwent *in vitro* chondrogenic differentiation proliferated rapidly and formed spheroids that increased in size over the course of the 3-week culture, being visible to the naked eye as early as day 3 after chondrogenic induction. The differentiated cells were positively stained by Alcian blue, specific for the glycosaminoglycans in cartilage matrix. The Ad-MSCs from both SJL and C57 strains showed similar potentials to differentiate to chondrocytes at passages 7 and 15 (Figure 4C). Differences were not apparent when compared with the chondrogenic differentiation of the C57-AdMSC population in any of its passages, demonstrating, as previously, the *bona fide* multipotent mesenchymal nature of the isolated SJL-AdMSCs.

### Clinical and neuropathological effects of adipose tissue-derived MSC therapy

**Figure 5:**
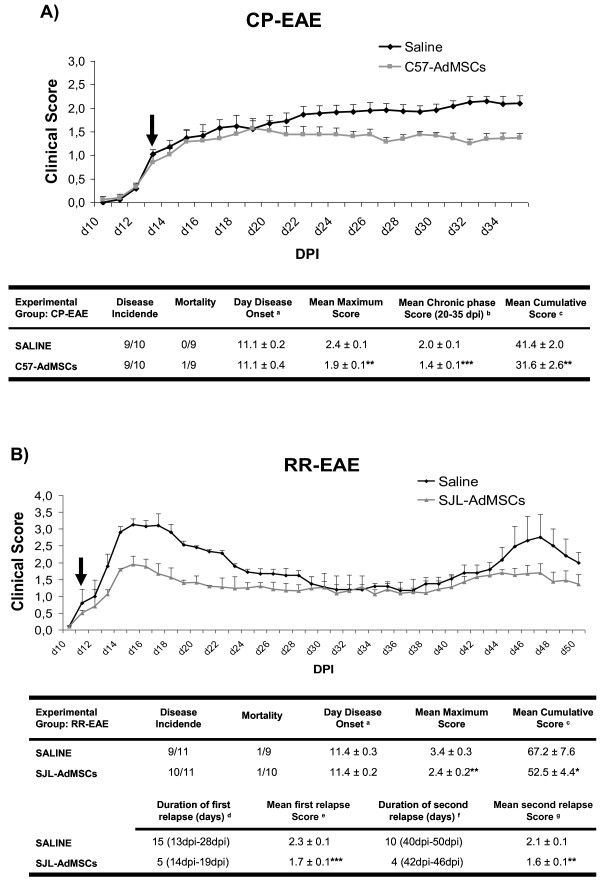
**Clinical outcome of experimental autoimmune encephalomyelitis models. (A)** Chronic progressive experimental autoimmune encephalomyelitis (CP-EAE) and **(B)** relapsing–remitting experimental autoimmune encephalomyelitis (RR-EAE) mice treated with C57-AdMSCs and SJL-AdMSCs, respectively. Graphs show the clinical score progression of each EAE model over the experimental period. Black arrows point to the day at which the treatment started. In the tables, the values are presented as mean ± standard error of the mean. Statistical analysis to perform single comparisons was carried out using Student’s *t* test. *P <0.05, **P <0.01, ***P <0.0001 vs. saline. ^a^Day disease onset, first day on which animals show any clinical symptoms (clinical score ≥0.5). ^b^Mean chronic phase score, mean EAE score from each experimental group over the chronic phase in CP-model (from 20 to 35 dpi). ^c^Mean cumulative score, average of the accumulated EAE score from each mouse over the entire experiment (until 35 dpi in CP-EAE and until 50 dpi in RR-EAE). ^d,f^Duration of first/second relapse, days of the first/second relapse. The beginning of the relapse was established when the animals had a clinical score of 1.5 or higher, and the end of that period arrived when the score recovered that value. ^e,g^Mean of first/second relapse score, average of the EAE score from each mouse over the mentioned relapsing periods. Ad-MSC, adipose tissue-derived mesenchymal stem cell; dpi, days post immunization.

The results showed that CP-EAE was developed in C57Bl/6-EAE-induced mice with a 90% of the disease incidence (Figure 5A). The first clinical signs of EAE appeared at 11.1 ± 0.2 dpi (mean ± SEM). Control mice, treated intravenously with saline at 13 dpi, suffered increased neurological deficit over time, and later presented a stable disease course, typical of this chronic model. Mice treated with C57-AdMSCs at the same time, showed a decrease in the severity of the disease, as reflected in the drastic reduction of the mean maximum (1.9 ± 0.1 vs. 2.4 ± 0.1 in control mice; *P* <0.01) and cumulative scores (31.6 ± 2.6 vs. 41.4 ± 2.0 in control mice; *P* <0.01) through the disease course. Furthermore, in these mice the chronic phase was established with a moderate symptomatology, the mean clinical score being significantly reduced when compared with control animals (1.4 ± 0.1 vs. 2.0 ± 0.1; *P* <0.0001) over this phase.

The MS RR-EAE model used in this study is characterized by a relapsing–remitting type of neurological deficit. The clinical course of this model is characterized by the appearance of at least two relapses separated by a period of symptomatic remission (Figure 5B). The onset of each relapse was considered when animals had a clinical score of 1.5 or higher, and the remission was regarded as the period in which the animals had 1.4 or less. Most of the SJL/JCrl immunized animals developed symptoms with no significant differences at the day of the disease onset between the experimental groups (11.4 dpi). Saline-treated mice showed a very aggressive multiphasic disease progression, as reflected by the highest maximum and cumulative mean scores. By contrast, RR-EAE animals that received a unique dose of SJL-AdMSCs at 12 dpi, presented a moderate clinical course with a significantly reduced maximum (2.4 ± 0.2 vs. 3.4 ± 0.3; *P* <0.01) and cumulative (52.5 ± 4.4 vs. 67.2 ± 7.6; *P* <0.05) mean scores. Moreover, the autologous transplants were able to reduce not only the duration of the relapses but also the severity of the neurological deficit suffered by the mice in these periods. Whereas control mice experienced a first 15-day relapse with a mean score of 2.3 ± 0.1, cell-treated animals reached a mean value of 1.7 ± 0.1 (*P* <0.0001 vs. saline) in a first relapse that only lasted 5 days. Although the second relapse was less aggressive than the first, cell therapy induced a significant decrease of both the aggressiveness (1.6 ± 0.1 vs. 2.1 ± 0.1; *P* <0.01) and the duration (4 vs. 10 days) of this relapse period when compared with the animals treated with saline.

Finally, we evaluated the correlation of observed clinical score amelioration with a reduction of the neurohistopathological damage. In order to do this, and according to the EAE clinical course, spinal cord processing was performed at chronification (35 dpi) and second relapse (45 dpi), in CP-EAE and RR-EAE animals, respectively.

**Figure 6:**
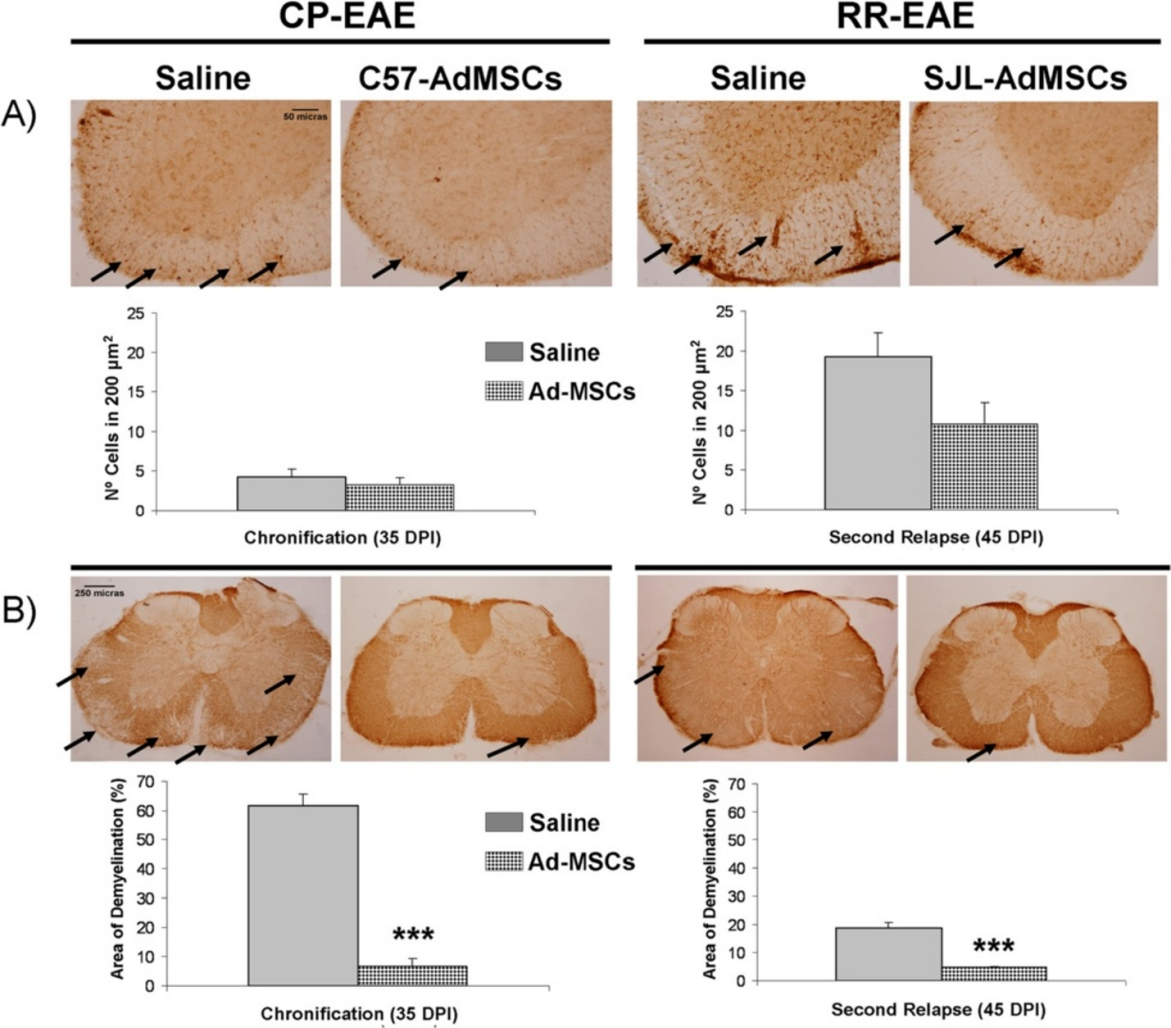
**Cell therapy effects in inflammatory infiltrates and demyelination in the spinal cord of experimental autoimmune encephalomyelitis mice.** Representative lumbar spinal cord sections from experimental autoimmune encephalomyelitis (EAE) mice, stained with anti-CD3 or anti-myelin basic protein (anti-MBP) antibodies to detect inflammatory infiltrates **(A)** and the degree of demyelination **(B)** in both chronic progressive experimental autoimmune encephalomyelitis (CP-EAE)-induced and relapsing–remitting experimental autoimmune encephalomyelitis (RR-EAE)-induced animals. Magnification: **(A)** 10× and **(B)** 4×. Graphs show the mean ± standard error of the mean of the number of T cells stained with CD3 in 200 μm^2^ **(A)**, or the demyelinated area, characterized by a lack of MBP staining related to the total area **(B)**, from three sections of six animals per each experimental group. *t* test: ***P <0.0005 vs. saline. Ad-MSC, adipose tissue-derived mesenchymal stem cell; DPI, days post immunization.

As expected, immunohistochemistry staining of spinal cord sections from chronified EAE saline-treated mice (CP-EAE) showed a minor presence of cell infiltrates accompanied by a considerable percentage of demyelination. No differences were found in T-cell infiltration between CP-EAE mice, irrespective the treatment received (Figure 6A) (4.2 ± 1.0 vs. 3.2 ± 0.9). The effect of the treatment, however, was very noticeable on the percentage of the total demyelinated area, assessed by myelin basic protein expression (Figure 6B). The autologous transplants of Ad-MSCs thus significantly inhibited the demyelination in the white matter of the lumbar spinal cord (61.7 ± 4.0 vs. 6.8 ± 2.5; *P* <0.0001 treatment vs. saline).

Similarly, cell therapy-mediated improvement of the EAE clinical course associates with neurohistopathological changes in RR-EAE mice. Consistent with the clinical data, a reduction of the number of CD3-positive cells in RR-EAE-induced animals was detected when treated with autologous cell therapy (Figure 6A), although no statistical differences were found when compared with animals treated with saline (19.2 ± 3.1 vs. 10.8 ± 2.7). Autologous Ad-MSC treatment also reduced significantly the percentage of demyelination in the white matter area of the lumbar spinal cord (18.6 ± 2.1 vs. 4.9 ± 0.2; *P* <0.0001 vs. saline), regardless of its minor incidence (Figure 6B).

## Discussion

Preclinical studies on MSCs, including Ad-MSCs, have allowed the cells to be considered as cell-based therapeutic strategies in regenerative medicine and autoimmune diseases. However, discrepancies reported from different authors regarding many biological features of stem cells must be solved before translating stem cell knowledge into clinical setting. The most contradictory results are due to different laboratory procedures and protocols, such as the tissue for obtaining MSCs, isolation methods and culture conditions, among others [9, 12–15, 19–21]. Different researchers also employ different animal species/strains whose genetic background make them more suitable to be used as a specific disease model, focusing their efforts on establishing MSC autologous transplantations as beneficial therapies in these diseases. The identification and characterization of any possible variability factors intrinsic to the animal model or cell source in cell-based approaches is mandatory in order to rule out false-positive results and to validate therapies. For all of these reasons, we thought it would be useful to compare the Ad-MSCs derived from different mice strains.

Specifically, we provide comparative data regarding the characteristics of Ad-MSCs from two inbred strains of mice, SJL/JCrl and C57BL/6, commonly employed to develop animal models for a variety of diseases [25, 28, 35–37]. Our data represent a pooling of Ad-MSCs from several animals of each strain, specially focused on interstrain differences and not on intrastrain differences, assuming from our own observations that the latter are minimal.

The results showed that Ad-MSCs isolated from SJL/JCrl mice (SJL-AdMSCs) displayed an analogous morphology and volume to those of the extensively studied Ad-MSCs isolated from C57BL/6 mice (C57-AdMSCs) when cultured for a long period of time under our experimental conditions. These results are in accordance with those explaining that MSCs are fibroblastoid-like cells enriched to homogeneity during a long-term culture [13, 45], persisting only with minimal alterations in cell morphology (size and shape) [22, 46].

The Ad-MSC growth rate increased through different culture passages, reaching its higher values after passage 7 for SJL-AdMSCs and after passage 6 for the C57-AdMSC population, and the growth stabilized at those values up to passage 15. These data suggest that, as well as the absence of morphological evidence of cell aging (distended or irregular flat cell shapes and more circumscribed nuclei under phase contrast microscopy), neither SJL-AdMSCs nor C57-AdMSCs undergo senescence phenomena at the highest passages evaluated. Our results are in agreement with previous studies in which they have maintained a prolonged *in vitro* expansion of murine MSCs, postulating that these cells, given the appropriate conditions, will remain and proliferate in culture without decreasing their growth rate [13, 19, 22]. However, although we find no evidence of senescence or slowing of growth with time, we cannot exclude that different experimental approaches could further influence their behavior. Previous works have thus reported evidence of senescent features under specific circumstances – that is, enlarged and irregular cell shapes and ultimately a stop of proliferation – demonstrating that many relevant factors play an important role in MSC expansion, such as different culture times and conditions, the tissue source from which MSCs are obtained, cell isolation protocols or cell density of the starting culture [14–17, 19, 22].

The SJL-AdMSC phenotype profile exhibited the lack of expression of hematopoietic markers in all of the passages evaluated. This result was very similar to that of C57-AdMSCs and is in agreement with those described in MSCs studies [9, 14, 15]. A variable expression level of stromal markers was detected in the SJL-AdMSC population, with no differences when compared with C57-AdMSCs. Hence, there is general consensus that the broad range of expression levels of these markers in MSCs is mainly attributed to the MSC tissue source, a specific culture and the experimental conditions [5, 7, 13, 15, 17, 18].

The multipotency of the SJL-AdMSC population at passages 7 and 15 was evaluated by *in vitro* differentiation into mesodermic origin cell lineages. We conducted these assays at those passages to ensure that the differentiation capacity was not lost at the highest passages. As we expected, SJL-AdMSCs were differentiated into adipocytes, osteoblasts and chondrocytes in both passages tested, with no differences when compared with C57-AdMSCs, indicating again their MSC condition.

Finally, we wanted to determine whether SJL-AdMSCs also exhibit the *in vivo* immunomodulatory properties described for C57-AdMSCs when transplanted into animal models of autoimmune diseases, as EAE [4, 40]. The EAE model plays an important role as a first-line model system in MS research. The EAE model is characterized by central nervous system inflammation and demyelination, like MS. Because there is no single EAE model that mimics the broad symptomatic variability in MS, we performed the autologous cell therapy in two frequently used EAE models in mice, RR-EAE and CP EAE, covering the clinical courses that appear more frequently in MS patients. As mentioned above, C57BL/6 and SJL/JCrl mouse strains are genetically susceptible and appropriate to develop CP-EAE and RR-EAE, respectively [25, 35–39].

The autologous transplants were carried out with Ad-MSCs from passages 7 to 9, this being the smaller range of passages in which, in addition to complying with the requirements for consideration as multipotent mesenchymal cells, the growth rate of these cell populations was optimal for obtaining the cell number required for *in vivo* administration. Moreover, the cell administration in lower passages might also avoid the chromosomal alterations described for Ad-MSCs after multiple divisions [13, 18].

Our results showed that the autologous administration of murine C57-AdMSCs after CP-EAE onset successfully ameliorated the severity of the disease. Clinical efficacy was demonstrated by a decreasing mean maximum and the cumulative disease score, as well as in a lowering of the clinical score in which the disease becomes chronic and stable. As expected, immunohistological staining of lumbar spinal cords showed a reduction of spinal cord inflammation as well as of demyelinated area after cell transplant. These results are consistent with others published previously [4, 40, 43].

As far as we know, this is the first study that describes the *in vivo* efficacy of SJL-AdMSCs when transplanted in RR-EAE induced mice. Gerdoni and colleagues reported the immunomodulatory properties of bone marrow MSCs isolated from C57BL/6 mice into the SJL-RR-EAE model [47]; however, no one has presented a description of or the preclinical study of the autologous transplantation of SJL-AdMSCs in the RR-EAE model that we present here. Results showed that symptoms in both the first and the second relapses lasted less time in transplanted animals than in those treated with saline, and the mean score reached over these periods was significantly reduced when compared with control mice. Cell transplantation significantly reduced the maximum and the cumulative score, inducing a less aggressive EAE in animals. These findings corroborate the clinical efficacy of Ad-MSC in EAE models.

Interestingly, neuropathological analysis confirmed that clinical amelioration was accompanied by a reduction of both central inflammation and demyelination in SJL-AdMSCs treated animals in comparison with control mice. These results suggest that this therapy could play an important role in counteracting the inflammatory processes occurring during relapse periods, and in neutralizing the neurodegeneration process, characteristic of the long-term progression of this RR-EAE model [39].

It is important to note that CP-EAE and RR-EAE models cannot be compared due to the various pathological processes determined by the different clinical courses, as demonstrated by significant differences in data concerning the number of T cells infiltrated and the total demyelinated area obtained from control animals. Notwithstanding, we could establish that autologous Ad-MSC transplantation is effective in modulating both models, possibly through mechanisms mainly based on immunomodulation and neuroprotection, as demonstrated in this work and by others.

## Conclusions

Our results demonstrate that isolated SJL-AdMSCs share *in vitro* mesenchymal similarities with standardized C57-AdMSCs, suggesting that the former might serve as an experimental model for many studies in the same way as the latter. In our hands, Ad-MSC populations tested at different culture passages, are appropriate for their experimental use, since cells fulfill the optimal morphology and phenotypic profile of MSCs, showing the highest and most stable growth rate, maintaining their multipotency and presenting immunomodulatory effects.

Most importantly, our data suggest that the SJL/JCrl mouse inbred strain might be a very suitable source of Ad-MSCs (as well as C57BL/6 mice) for preclinical studies related to the Ad-MSC biological properties and their application as promising therapeutic tools in experimental medicine for autologous transplantations.

Other approaches are required to evaluate whether these cells maintain a normal karyotype in the suggested culture passages. Moreover, additional studies aimed to establish a standard model for murine Ad-MSC expansion and to unify the criteria of the scientific community are warranted.

## Abbreviations

Ad-MSC: adipose tissue-derived mesenchymal stem cell
C57-AdMSCs: adipose tissue-derived mesenchymal stem cells obtained from the C57BL/6 mouse inbred strain
CP-EAE: chronic progressive experimental autoimmune encephalomyelitis
dpi: days post immunization
*DT*: doubling time
EAE: experimental autoimmune encephalomyelitis
MS: multiple sclerosis
MSC: mesenchymal stem cell
PFA: paraformaldehyde
RR-EAE: relapsing–remitting experimental autoimmune encephalomyelitis
SEM: standard error of the mean
SJL-AdMSCs: adipose tissue-derived mesenchymal stem cells obtained from the SJL/JCrl mouse inbred strain.
